# Reactive Oxygen Species and Metabolism in Leukemia: A Dangerous Liaison

**DOI:** 10.3389/fimmu.2022.889875

**Published:** 2022-06-09

**Authors:** Marta Romo-González, Carla Ijurko, Ángel Hernández-Hernández

**Affiliations:** ^1^ Departamento de Bioquímica y Biología Molecular, Universidad de Salamanca, Salamanca, Spain; ^2^ Instituto de Investigación Biomédica de Salamanca (IBSAL), Hospital Universitario de Salamanca, Salamanca, Spain

**Keywords:** reactive oxygen species, metabolism, leukemia, NADPH oxidases (NOX), hematopoietic stem cell (HSC), leukemic stem cell (LSC)

## Abstract

Reactive oxygen species (ROS), previously considered toxic by-products of aerobic metabolism, are increasingly recognized as regulators of cellular signaling. Keeping ROS levels low is essential to safeguard the self-renewal capacity of hematopoietic stem cells (HSC). HSC reside in a hypoxic environment and have been shown to be highly dependent on the glycolytic pathway to meet their energy requirements. However, when the differentiation machinery is activated, there is an essential enhancement of ROS together with a metabolic shift toward oxidative metabolism. Initiating and sustaining leukemia depend on the activity of leukemic stem cells (LSC). LSC also show low ROS levels, but unlike HSC, LSC rely on oxygen to meet their metabolic energetic requirements through mitochondrial respiration. In contrast, leukemic blasts show high ROS levels and great metabolic plasticity, both of which seem to sustain their invasiveness. Oxidative stress and metabolism rewiring are recognized as hallmarks of cancer that are intimately intermingled. Here we present a detailed overview of these two features, sustained at different levels, that support a two-way relationship in leukemia. Modifying ROS levels and targeting metabolism are interesting therapeutic approaches. Therefore, we provide the most recent evidence on the modulation of oxidative stress and metabolism as a suitable anti-leukemic approach.

## Introduction

Leukemia is an abnormal proliferation of white blood cells that occurs in bone marrow (BM) and expands through the blood. It is initiated by a hematopoietic progenitor cell that suffers one or more malignant transformations, which are attributed to genetic and environmental factors. It can be classified as acute or chronic, depending on the speed of spreading, or as myeloid or lymphoid, depending on the lineage of the transformed cells. Thus, the main types of leukemia are:

1. Acute Myeloid Leukemia (AML): It is the most diagnosed leukemia in adults. It is characterized by a very heterogeneous molecular landscape that impairs its treatment what results in a 5 years-overall survival rate of 29.5% (1).
2. Acute Lymphoid Leukemia (ALL): It is prevalently detected in younger people; the median diagnosis age is 17. Good prognosis is expected for patients under 20 whereas most of deaths occur in older patients (2).
3. Chronic Myeloid Leukemia (CML): It is occasioned by t (9;22), named as Philadelphia chromosome, that provokes the generation of an oncokinase (BCR-ABL) that triggers the neoplasia. Targeting BCR-ABL started in 2001 and has improved the CML outcome extraordinarily.
4. Chronic Lymphoid Leukemia (CLL): It is considered as a disease of older adults with a median age of 70 at diagnosis. Molecular characterization of the pathology has rendered the opportunity of developing new drugs against key targets such as BTK, BCL-2 and PI3K which application has increased the 5-year overall survival up to 86% (3).

As recently reported, the global incidence of leukemia is 474,519 cases worldwide, which implies 11 cases per 100,000 inhabitants (4), with an age-standardized incidence rate of 6.76/100,000 (95% uncertainty interval 6.15 to 7.16) (5). Subsequently, latest prevalence data of the disease point to 2.43 million of patients worldwide, with an age-standardized prevalence rate of 32.26 per 100,000 (95% uncertainty interval 29.02 to 34.61) (6). The mortality rate of leukemia is 3.2 (4), that standardized by age result in 4.50 (95% uncertainty interval 4.12 to 4.73) (6). Leukemia is more prevalent in males and in countries with higher socio-demographic development (6). Studies of indicators’ evolution from 1997 to 2017 reflect improvements in terms of disability-adjusted life years (DALYs), incidence and mortality but are minimal (6), what couples with an increasing worrying prevalence. Therefore, the study of leukemia remains a priority. In this review, we discuss two features, redox regulation and metabolism, that affect the transformation from non-pathological to leukemic status.

## Reactive Oxygen Species (ROS) Levels Matter in Hematopoiesis

Researchers have accepted the ability of ROS to regulate cellular signaling. It has long been known that the addition of exogenous hydrogen peroxide (H_2_O_2_) stimulates cell proliferation (7). This explains why signaling through certain cytokines and growth factors is accompanied by ROS production (8, 9). The list of signaling proteins that can be regulated by reversible oxidation is expanding (10), suggesting the relevance of redox signaling for cell biology.

There is a plethora of evidence supporting the importance of redox signaling in the regulation of cell differentiation, including hematopoiesis (11, 12). The correct production of all mature blood lineages relies on the potential of hematopoietic stem cells (HSC). HSC reside in the BM, where a strict balance between self-renewal and differentiation must be maintained (13).

HSC show low ROS levels, which seem to be required to maintain their self-renewal and stemness capacity (14). Serial transplantation experiments have shown that endogenous high ROS levels are detrimental to HSC function (15). Moreover, extraphysiologically induced oxidative stress impairs the HSC reconstitution capacity (16). These findings support the notion that ROS levels need to remain low to preserve HSC functionality. Such a fine balance depends on extrinsic and intrinsic factors.

As reviewed elsewhere (12), gene targeting experiments have revealed certain key genes in maintaining low ROS levels in HSC, such as FoxO transcription factors (17), tuberous sclerosis complex 1 (*Tsc1*) (18), ataxia–telangiectasia mutated gene (*Atm*) (19), and polycomb group (PcG) *Bmi1* (20). These studies have revealed a common pattern of loss of HSC functionality linked to an increase in ROS levels. The fact that in some of these reports, antioxidant treatment could rescue HSC defects strongly suggests the detrimental effect of oxidative stress on HSC homeostasis. Enhanced ROS production in HSC could induce DNA damage, leading to premature senescence and, eventually, to loss of stem cell properties (21).

Adult HSC reside and develop in the BM, where their environment or niche has a prime role in regulating HSC biology. Among the extrinsic factors that help to maintain low ROS levels in HSC, the influence of the niche is probably the most important. Defining the niche is complicated, and not so long ago a perivascular and endosteal niche were considered to exist. However, recent advances suggest that the niche is mainly perivascular and composed of mesenchymal stromal cells and endothelial cells (22). Despite being highly vascular, the oxygen concentration in the BM is rather low, with the most hypoxic environment in the peri-sinusoidal regions (23). This environment would favor HSC living under hypoxic conditions and thereby with low ROS levels. In addition, niche cells can protect from oxidative stress *via* ROS uptake from HSC (24). Some reports suggest that stabilization of the transcription factors hypoxia-inducible factor 1-alpha (HIF1α) and 2-alpha (HIF2α) under these circumstances is required to regulate HSC homeostasis (25, 26). In contrast, other authors maintain that the lack of HIF1/2α does not affect the adult HSC self-renewal capacity (27, 28). Signals from the niche could also contribute to keep ROS levels low. CXC chemokine ligand 12 (CXCL12) signaling through its receptor CXCR4 is one axis that contributes to maintain HSC quiescence, which is achieved, at least in part, by keeping ROS levels low (29).

ROS levels may influence signaling pathways that are key to control the balance between quiescence and proliferation of HSC. Activation of the phosphoinositide 3-kinase (PI3K)/AKT and mitogen-activated protein kinase (MAPK) pathways is required for HSC proliferation (30); these signaling pathways can be redox regulated and simultaneously contribute to ROS production. AKT behaves as coordinator of a number of different signaling pathways, and it is a crucial regulator of HSC homeostasis. AKT1 and AKT2 deletion is associated with a significant decrease in ROS levels and excessive quiescence that impairs the HSC reconstitution ability (31). Alternatively, constitutive activation of AKT increases HSC cycling, leading to leukemogenesis (32). It has been well documented that constitutive activation of the mitogen-activated protein kinase kinase (MEK)/extracellular signal-regulated kinase (ERK) pathway is required for megakaryocytic differentiation depends on ROS production (33). In contrast, activation of p38 MAPK in response to oxidative stress impairs HSC function (34).

A sustained HSC activation induced by signaling pathways such ERK and PI3K can lead to their exhaustion due to an excessive mTOR-induced mitochondrial ROS production, which damage these organelles. Keeping HSC homeostasis depends on the existence of counterbalance mechanisms. One of such feedback processes is the phosphorylation of MEK1 by ERK, that limits the activation of the PI3K/AKT/mTORC1 pathway (35). Additionally, it has been shown that retinoic acid signaling can also contribute to keep HSC quiescence, among other reasons, by limiting ROS production (36).

This evidence illustrates the importance of ROS levels in regulating HSC functionality.

## ROS Production Is Increased in Leukemia

In agreement with the original perception of ROS as harmful by-products, tumor cells display a high ROS levels (12). Oxidative DNA damage and an enhanced mutation rate could result from the involvement of ROS in tumor transformation. This notion has been supported for a long time, since the original experiments showing that H_2_O_2_ could induce activating mutations of the *RAS* oncogene (37), or inactivating mutations of *TP53* tumor suppressor (38). On the other hand, ROS scavenging can limit cell transformation (39). Tumor cells can harness their high ROS levels as an advantage to promote cell proliferation through the activation of signaling cascades such as MAPK (40). In addition, ROS activate survival pathways such as PI3K/AKT signaling, allowing tumor cells to evade apoptosis (41). Oxidation and inactivation of protein and lipid phosphatases may pave the way to activation of the above-mentioned signaling pathways. Finally, oxidative stress has been related to chemotherapy resistance (42), which makes ROS an attractive target for overcoming multidrug resistance (43).

Hematological malignancies are not strangers to this scenario, with reports specifying elevated ROS levels in chronic myeloid leukemia (CML) (44) and acute myeloid leukemia (AML) (45). It is assumed that ROS contribute to myeloid cell transformation (46), and to maintaining cell proliferation (47). More recently, oxidative stress has been related to chronic lymphocytic leukemia (CLL) (48), acute lymphocytic leukemia (ALL) (49), and T-cell acute lymphoblastic leukemia (T-ALL) (50).

The causes explaining oxidative stress in leukemic cells can be diverse, including activation of ROS production by oncogene signaling. *BCR-ABL* is the prototypical leukemia-driving oncogene. BCR-ABL triggers ROS production, leading to an enhanced rate of mutagenesis and genetic instability (51). FLT3-ITD, another kinase involved in AML, could play a similar role (52). *RAS* mutations occur frequently in leukemia, and are commonly associated with a dismal prognosis. Enhanced ROS production has been reported in hematological patients with *RAS* mutation (53). *JAK2* activating mutations are found in different hematological neoplasia. Recent reports support the importance of ROS in leukemic process induced by JAK2 hyperactivation (54).

Reduced activity of several antioxidant enzymes has been reported in CML (55), AML (56), ALL (57), and CLL (58). Therefore, a defective antioxidant barrier could also be involved in the generation of oxidative stress in leukemia.

Finally, the most straightforward way of explaining oxidative stress in leukemia is through enhanced ROS production by any of the two main sources cellular ROS: the mitochondria and the NADPH oxidase family. NADPH oxidases are a family of membrane-bound enzymes whose only known function is ROS production. The founding member is the phagocyte oxidase, which is required for innate immunity (59). It had been thought that this ROS-producing system only occurred in myeloid cells. However, cloning the first homolog of the catalytic subunit of the phagocyte oxidase in 1999 (60) led to the discovery of a family comprising seven isoforms (NOX1, NOX2, NOX3, NOX4, NOX5, DUOX1, and DUOX2). These members differ in their expression pattern and their activation requirements (61). NADPH oxidases are present in all eukaryotic cells, and it is common to find several isoforms in each type of cell, a finding that strongly suggests an indispensable role in cellular biology. This is the case for HSC, which express different NAPDH oxidase isoforms (62, 63), and recent reports support their relevance in the control of HSC biology and differentiation (64–66).

Compelling evidence support the relevance of NADPH oxidases in the enhanced ROS production that occurs in different types of leukemia such as AML (45), immunoresistant CML (67), chronic myelomonocytic leukemia (68), hairy cell leukemia (69), and adult T-cell leukemia induced by the human T-cell leukemia virus type 1 (70). Bearing in mind that oxidative stress in leukemic cells can be triggered by oncogene signaling, these enzymes could be one of the main targets of oncogenes in leukemia. In agreement with this notion, increased ROS production through NADPH oxidases has been associated with BCR-ABL (71), FLT3-ITD (72), and RAS (73). In this scenario, NOX2, the founding member of the family, has been associated with the regulation of the self-renewal of leukemic stem cells (64) in AML, leukemia cells expansion (74), and immunoresistant CML (67).

Enhanced mitochondrial ROS production has been also reported for some leukemic cells, such CLL (48). It has been suggested that mitochondrial ROS production enhances genomic instability in leukemic stem cells (LSC), allowing CML progression (44). Although additional studies are needed to fully understand the precise relevance of mitochondrial ROS in the leukemic process, damage to mitochondrial DNA (mtDNA) by oxidative stress may be a key event. mtDNA is not packed in chromatin, which makes it more susceptible to oxidative damage than nuclear DNA. Moreover, the lack of introns in mtDNA enhances the chances that mutations alter the genetic message. In line with this notion, mutations in mtDNA have been reported in CML (75), AML (76), CLL (77), and ALL (78).

## Metabolism in Leukemia: Not as Simple as Originally Thought

**Figure 1:**
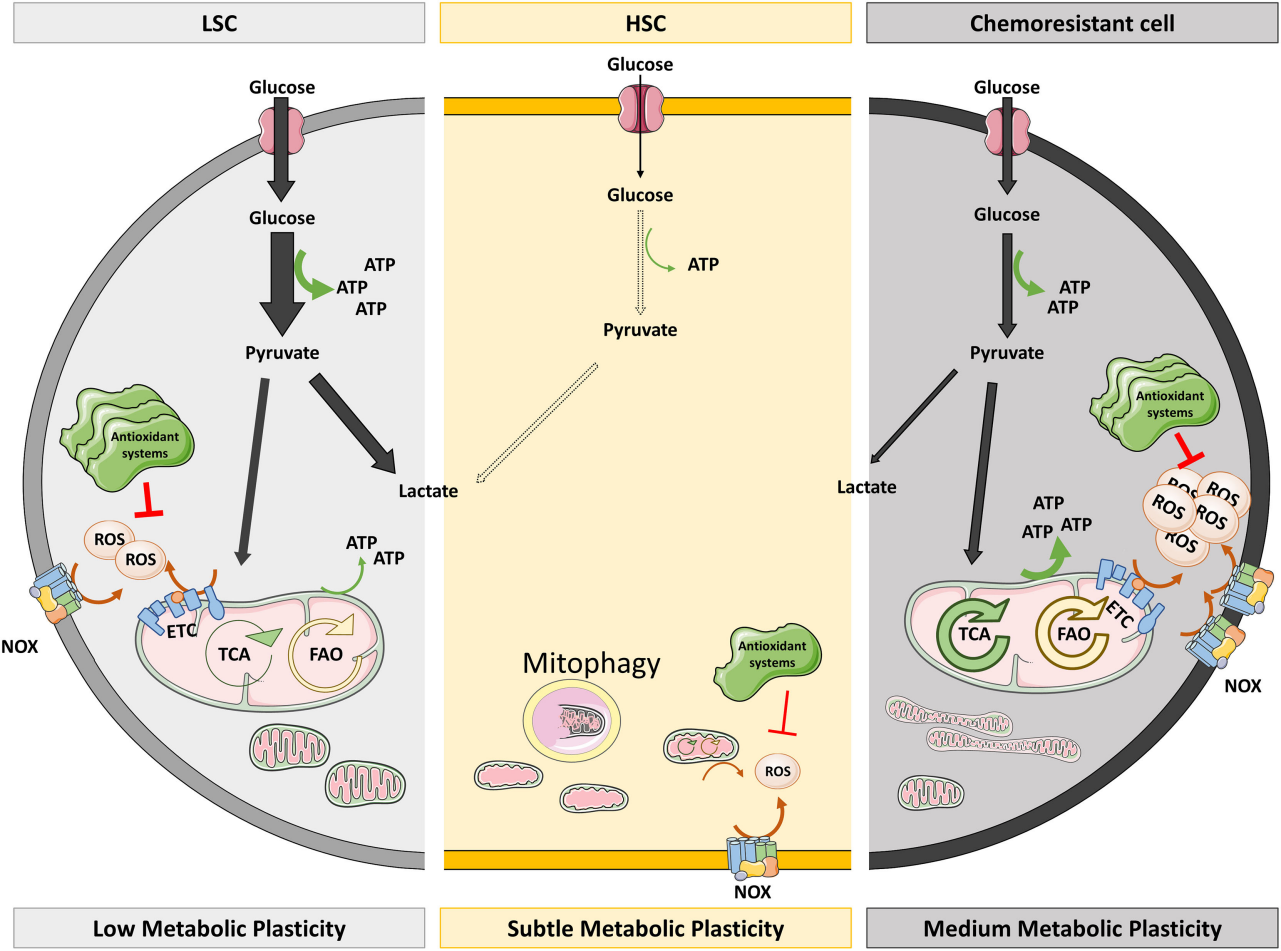
Redox and metabolic status of HSC versus LSC and chemoresistant cells. HSC are characterized by low metabolic activity and ROS levels. The low ATP levels they need come from glycolysis as they have immature mitochondria which suffer mitophagy. Low NOX levels and mitochondrial metabolism could explain the low ROS levels of HSC, which do not require high antioxidant systems to keep them under control. However, LSC actively obtain energy mainly by glycolysis (thick arrow) but also from TCA and FAO (thin arrows). This active metabolism provokes the production of ROS that are quenched by overexpressed antioxidant systems. On the right side of the figure, chemoresistant cells also produce ATP by glycolysis although they show mature and fused mitochondria which render high OXPHOS, being fed by hyperactivated TCA and FAO. Augmented OXPHOS and NOX result in high ROS levels which cannot be extinguished despite high antioxidant systems. ETC, electron transport chain; FAO, fatty acid oxidation; HSC, hematopoietic stem cells; LSC, leukemic stem cells; NOX, NADPH oxidase; OXPHOS, oxidative phosphorylation; TCA, tricarboxylic acid.

As has been discussed thoroughly, HSC should maintain tight control of ROS because low levels of these molecules are critical to maintain quiescence, differentiation, proliferation, and survival processes. HSC emerge from the aorta-gonad-mesonephros mesoderm during embryonic development and move to the fetal liver, undergo an expansion process, and finally settle in the BM, where they spend adult life. For the vast majority of their existence, HSC must loiter in quiescence, a state necessarily governed by low ROS levels. To this end, glycolytic metabolism is surmised to be the most appropriate energy source to these cells because its alternative, mitochondrial metabolism, is known as the main ROS producer in cells. Glycolysis meets all the requirements needed by HSC: low energy demand, unparalleled adaptability to a niche, and diminished ROS production due to the non-utilization of the electron transport chain (ETC). For a long time, HSC were considered to have low mitochondrial content. It has been shown that mitochondrial content of multi-potent progenitor cells (MPP) and HSC is similar, but the respiratory capacity of MPP exceeds that of HSC (79). One possible explanation is that HSC are exposed to a high mitochondrial turnover and mitophagy, but both are comparatively low in HSC. New evidence suggests that HSC present more immature mitochondria subjected to an intricate lysosomal degradation (80, 81). How mitochondrial content and activity is regulated in HSC is an important question to be solved in the future. Interestingly, researchers have identified HSC subpopulations with different metabolism. The latest approaches point to the existence of a metabolically inactive minority population of long-term HSC (approximately 25%) and a more abundant metabolically active population that obtains adenosine triphosphate (ATP) derived mainly from glycolysis but also from the tricarboxylic acid cycle (TCA)-ETC (81). Although HSC had been classified as glycolytic, researchers have begun to reveal the necessity of mitochondrial metabolism for HSC homeostasis (81, 82). Moreover, glycolytic restriction is required for HSC repopulation potency (81) (**Figure 1**). Many master regulators of metabolism such as liver kinase B1 (LKB1) (83), PI3K-AKT-mTOR (35), AMP-activated protein kinase (AMPK) (83), and protein tyrosine phosphatase mitochondrial 1 (PTPMT1) (84) have demonstrated an effect on the regulation of HSC fate decision *via* metabolic changes. Among these signaling pathways and others, the one most often shown as responsible for HSC metabolic fate are HIF1/2α (85). HIF1α deletion provokes the switch from glycolysis to oxidative phosphorylation (OXPHOS) on par with impairment of the reconstitution ability of HSC (86). Of note, researchers have recently questioned the involvement of HIF1/2α in HSC metabolic behavior, demonstrating that the control exerted by HIF1/2α over LSC metabolism is not decisive (87) and that HIF1/2α are not required for cell-autonomous HSC maintenance (27, 28).

**Figure 2:**
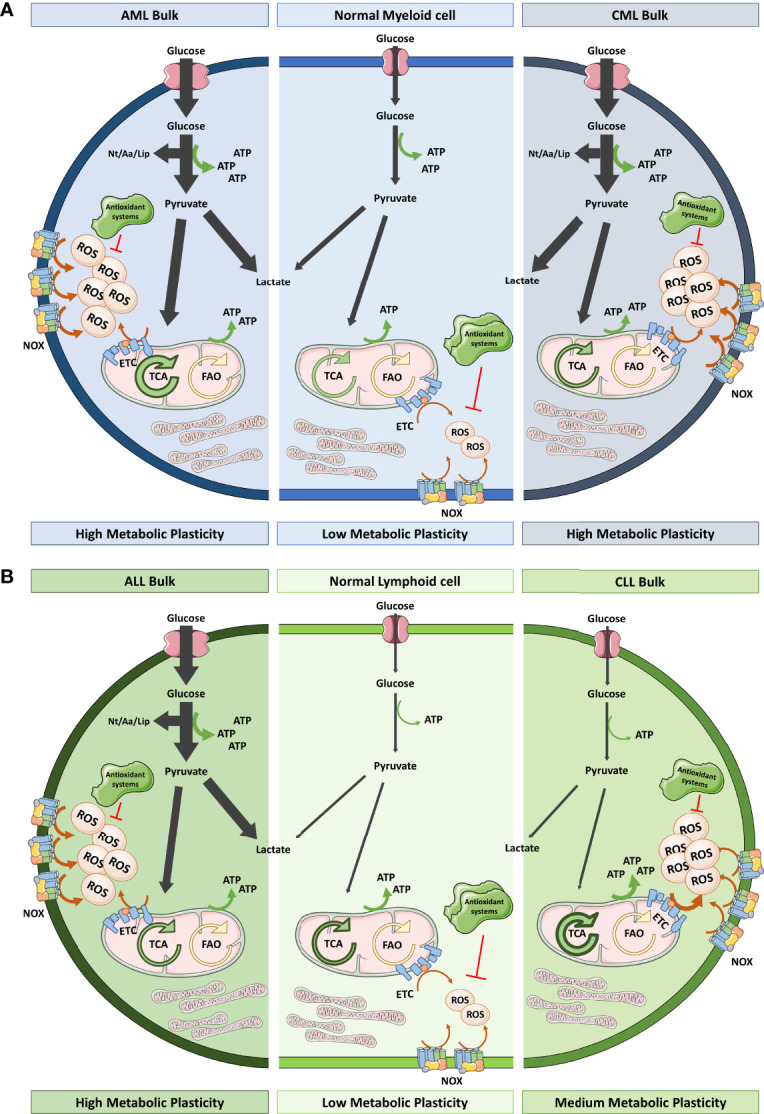
Redox and metabolic status of myeloid and lymphoid cells versus their leukemic counterparts. **(A)** Healthy myeloid cells obtain ATP from glycolysis and OXPHOS in a balanced way. They produce moderate ROS levels that could be variable depending on the differentiation status. However, AML bulk cells overstimulate glycolysis and TCA driven OXPHOS (thick arrows) to satisfy their proliferation needs in terms of energy and building blocks (Nt, Aa and Lip). This exacerbated metabolism together with the elevated NOX levels means that these cells are exposed to high oxidative stress. On the other hand, CML bulk cells rely more on glycolysis as BCR-ABL conditioned the metabolism towards aerobic fermentation. CML bulk cells contain elevated ROS levels coming from NOX and mitochondrial metabolism. Both, AML and CML, have been described as having low levels of antioxidant systems. **(B)** Healthy lymphoid cells hardly use glycolysis for energy obtaining instead they have an active TCA that triggers OXPHOS. ALL bulk cells have increased glycolytic flux comparing to their healthy counterparts whereas CLL bulk cells display TCA driven OXPHOS dependency for energy production. ALL and CLL bulk cells show high ROS levels as a result of high NOX expression, low antioxidant systems and, in the case of CLL, hyperactivation of ETC. Leukemic cells, irrespective of the lineage of origin, show notorious metabolic plasticity which permits their adaptation to different cellular stimuli and metabolic sources. Aa, amino acids; AML, acute myeloid leukemia; ALL, acute lymphocytic leukemia; CLL, chronic lymphocytic leukemia; CML, chronic myeloid leukemia; ETC, electron transport chain; FAO, fatty acid oxidation; Lip, lipids; NOX, NADPH oxidase; Nt, nucleotides; OXPHOS, oxidative phosphorylation; TCA, tricarboxylic acid.

During their lifetime, HSC undergo events in which they change their metabolism to OXPHOS: expansion in the fetal liver (86), proliferation (88), differentiation (84), aging (86), and tumor transformation. When utilizing OXPHOS, cells produce all the energy and the metabolic intermediates needed for the above-mentioned processes (88). The metabolic switch to OXPHOS increases ROS production and, consequently, most of these scenarios are characterized by high ROS levels. However, LSC must control their ROS levels more precisely, to maintain the benefits of increased OXPHOS without losing their quiescent potential. The main barrier against ROS in LSC is increasing the expression of antioxidant genes (89), although researchers have described that the BM microenvironment could transfer antioxidant molecules to LSC (90). Thus, LSC and HSC display distinct metabolic features (91) (**Figure 1**). First described by Laganidou (92), AML LSC—mainly quiescent with low ROS—resort to OXPHOS and are unable to use glycolysis when mitochondrial metabolism is inhibited. Moreover, they fuel the ETC with products from amino acid catabolism (93), rendering OXPHOS activity that is approximately three times higher than their counterparts (94) (**Figure 2A**).

Researchers had long believed that LSC were the cells responsible of relapse, but Farge et al. (95) recently described that neither LSC nor immature cells nor quiescent cells have been localized in the bulk of relapsed cells. The cells implicated in relapse develop a particular gene signature that activates OXPHOS, and together with an increased mitochondrial mass in their cytoplasm, result in ETC activation and high ROS levels. These cells rely on fatty acid oxidation to feed OXPHOS (93) and present elevated expression of CD36, a fatty acid transporter, as a tool to evade therapeutically induced apoptosis (95). The above information indicates that the OXPHOS dependence exerted by LSC is shared with chemoresistant cells but not with healthy HSC (**Figure 1**).

In addition to LSC, bulk cells are another population to be considered in leukemia. Based on current evidence, this large population mainly prefers lactic acid fermentation for energy production (96) (**Figure 2**). Discovered by Otto Warburg in 1931, most of the cancer cells display glycolytically enhanced metabolism even in the presence of sufficient oxygen, a state called aerobic fermentation. This pathway provides intermediates for their anabolic pathways, greater antioxidant protection, and greater ATP availably given that glycolysis occurs 100 times faster than OXPHOS. This metabolic switch is broadly accepted as a hallmark of cancer. Cheng et al. (97) described enhanced glycolysis in AML and went further by reporting a panel of six genes related to glycolysis that demonstrated prognostic value. However, Lo Presti et al. (98) recently described that patients with AML exhibit diverse metabolomes according to their cytogenetic and molecular alterations. Glycolysis is promoted in such cases in which leukemia is induced by oncokinases such as FLT3-ITD in AML (99) or BCR-ABL (100) in CML or ALL. However, in other typical translocations in leukemia—for example, MLL-AF9—OXPHOS occurs preferentially. That is also the case of CLL, which does not follow the Warburg effect (101) (**Figure 2B**). In fact, the general trend of cancer cells is to over-induce glycolysis while mitochondria continue to function as in the non-pathological conditions (102), which shows that cells still use mitochondrial metabolism and can resort to its excessive use if needed.

Continuing with the same example, AML cells, which have more mitochondria, have an impaired respiratory system due to lower spare respiratory capacity per mitochondrion that leads to ROS accumulation (103). Either way, we know that the contribution to ATP from the mitochondria of leukemic cells is similar to that of normal cells in most types of leukemia, except CLL (as previously mentioned) (**Figure 2**). Additional metabolomic studies are needed to describe the conditions that drive leukemic cells to choose which TCA source contributes most to this OXPHOS activity because both amino acid (104) and fatty acid (105) oxidation have been implicated, demonstrating a high adaptability of their metabolism to ambient conditions. Hence, this metabolic flexibility is starting to be depicted in all the populations that we previously described—bulk cells (106), LSC (107) and chemoresistant cells (108)—and it is important to consider because it hinders the possibility of using metabolism as a therapeutic target (109).

## ROS and Metabolism in Leukemia: A Two-Way Relationship

It is noteworthy that different oncogenic drivers, such as BCR-ABL, FLT3-ITD, or RAS, induce both an increase in ROS and metabolic rewiring in leukemic cells. Therefore, it can be surmised that both phenomena are related. Activation of redox signaling in this scenario could eventually induce metabolic changes during leukemic transformation. A way of testing such a hypothesis would be by checking whether the cellular sources of ROS involved in redox signaling affect metabolism.

NADPH oxidases again come into focus. Compelling evidence implicate several members of the family in metabolic regulation. It has been suggested that NOX4 functions as a mitochondrial sensor (110) that intervenes in the control of mitochondrial dynamics (111); it can also suppress the TCA cycle (112) and respiratory chain complex I (113). Other authors have suggested the importance of NOX4 in the induction of glycolysis through the activation of HIF1α (114). NOX1 has also been highlighted as a required participant in the metabolic redirection toward glycolysis (115). In addition, NOX2, the main isoform expressed in the hematopoietic system, has been related to metabolic control. A pioneering study conducted by Prata et al. (116) suggested that NOX2 and NOX4 are involved in glucose transport mediating GLUT1 hyperactivation. Later, Lu et al. (117) showed that NOX2 activity is required to maintain a high glycolytic rate in cancer cells upon inhibition of mitochondrial function. Baillet et al. (118) have shown that NOX2 activity is required to maintain hyperglycolysis in activated neutrophils. The authors described the co-localization of NOX2 and phosphofructokinase 2 (PFK2), and glycolytic activation depends on phosphorylation of the latter enzyme. Interestingly, the connection between NOX2 and metabolism also becomes apparent in the leukemic context. It seems that NOX2 is one of the main cellular sources responsible for the high ROS levels displayed by blast cells from patients with AML (45). Such elevated ROS levels seem to be required for the induction of glycolysis by upregulating the expression of the glycolytic enzyme 6-phosphofructo-2-kinase/fructose-2,6-bisphosphatase 3 (PFKFB3) (119). In addition, Adane et al. (64) have recently shown that NOX2 is required for LSC self-renewal. According to the authors, such a requirement implicates NOX2 in metabolic control. NOX2 deficiency rewires metabolism in leukemic cells, enhancing the use of fatty acids by the mitochondria and reducing the glycolytic flow that impairs the metabolic capacity of LSC. Moreover, we have recently demonstrated that AML patients show variable expression of NOX2 complex genes. NOX2 levels correlate with the expression of 28 metabolic genes, indicating that depending on the expression of this panel of genes (29G), patients with AML may show distinct metabolic patterns. In addition, 29G is capable of discriminating AML prognosis and survival, suggesting the relevance of metabolism in this type of leukemia (120). Finally, Marlein et al. (121) described an unexpected link between NOX2 and metabolism: superoxide produced by NOX2 drives the transfer of mitochondria from BM stromal cells to leukemic blasts, a phenomenon that could protect cells from chemotherapy (122). In summary, the metabolic changes observed during leukemic transformation might be under the control of redox signaling, and NADPH oxidases could be one of the sources of ROS involved in such a process.

Undoubtedly, the best-known metabolic regulation of ROS lies in their ability to control the expression or activity of metabolic master regulators such as HIF1α, AMPK, or ATM. AMPK is activated when the ATP supply is insufficient, in order to restore ATP. ROS are able to activate AMPK activity both directly (123) and indirectly (124). This reprograms cellular metabolism by promoting catabolic pathways that increase glucose uptake, glycolysis, fatty acid uptake and oxidation, and mitochondrial biogenesis, while it suppresses anabolic processes such as protein and lipid synthesis. As we described above, HIF1α is another redox-sensitive metabolic regulator. ROS promote dimerization and inactivation of prolyl hydroxylase domain (PHD) protein, resulting in stabilization of HIF1α (125). HIF1α increases the expression of multiple genes involved in glucose metabolism, including glucose transporters (GLUT1 and GLUT2), hexokinase (HK), PFKFB3, pyruvate kinase M2 (PKM2), and pyruvate dehydrogenase kinase (PDK) (126). Finally, we note that direct oxidation of certain cysteine residues of the ATM protein induces its dimerization leading to its activation (127). Although with a much less studied metabolic role, this DNA damage response kinase can promote the pentose phosphate pathway (PPP) by increasing glucose-6-phosphate dehydrogenase (G6PD) activity (128). Overall, activation of these three master regulators of metabolism favors the Warburg effect by generating a metabolic shift from mitochondrial OXPHOS to glycolysis and the PPP pathway. Regulation of the expression of genes encoding metabolic enzymes by such master regulators provides a means by which redox signaling could orchestrate a metabolic change in leukemic cells (129). Another possibility, somehow overlooked, is regulation of the activity of the key metabolic enzymes by redox mechanisms.

## Redox Regulation of Metabolic Enzymes

ROS can directly regulate the activity of multiple metabolic enzymes and thus control cellular metabolism. Among the ROS generated in the cell, H_2_O_2_ is the most relevant in redox signaling. This is due to its physicochemical properties compared with other oxyradicals: moderate reactivity, the ability to diffuse across membranes, and a long half-life. The most widely studied oxidative protein modification is cysteine thiol group (R-SH) oxidation, also known as S-oxidation. Oxidation of the thioester group of methionine, and oxidation of tyrosine and tryptophan residues with the capacity to modify enzyme activity, have also been described (130).

Despite the increased specificity provided by H_2_O_2_, many authors believe that it cannot be considered a true second messenger because it could interact anarchically with all thiol-susceptible proteins and modify their activity (131). Here is where S-glutathionylation comes into play, giving ROS true power as second messengers. However, given the lack of specific probes for different ROS, as well as the lability of these ligands, it is very difficult to determine which oxidation process is responsible for a modification in the function of a given enzyme (132).

**Figure 3:**
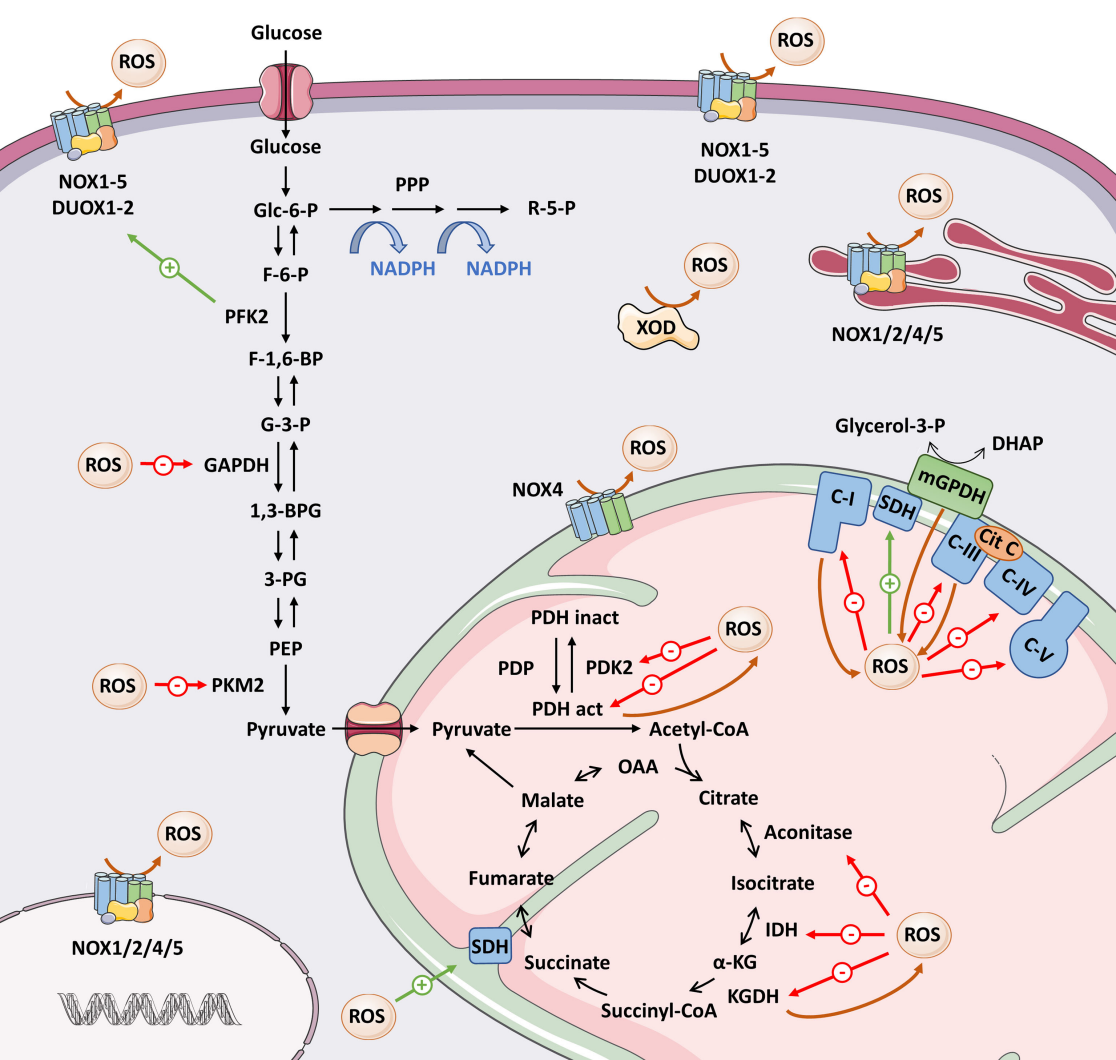
Overview of the interrelationship between ROS and metabolic enzymes. Here we present the main ROS-producing systems, as well as the metabolic enzymes whose activity is modified by direct oxidation (see text for details). Brown arrows mean ROS production; red arrows mean inhibition; green arrows mean activation of target. 1,3-BPG, 1,3-bisphosphoglycerate; 3-PG, 3-phosphoglycerate; Cit C, cytochrome C; DHAP, dihydroxyacetone phosphate; F-1,6-BP, fructose-1,6-bisphosphate; F-6-P, fructose-6-phosphate; G-3-P, glyceraldehyde-3-phosphate; GAPDH, glyceraldehyde-3-phosphate dehydrogenase; Glc-6-P, glucose-6-phosphate; IDH, isocitrate dehydrogenase; α-KG, α-ketoglutarate; KGDH, α-ketoglutarate dehydrogenase; mGPDH, mitochondrial α-glycerophosphate dehydrogenase; OAA, oxaloacetate; PDH act, pyruvate dehydrogenase complex active; PDH inact, pyruvate dehydrogenase complex inactive; PDK2, pyruvate dehydrogenase kinase 2; PDP, pyruvate dehydrogenase phosphatase; PEP, phosphoenolpyruvate; PFK2, phosphofructokinase 2; PKM2, pyruvate kinase M2; PPP, pentose phosphate pathway; R-5-P, ribose 5-phosphate; ROS, reactive oxygen species; SDH, succinate dehydrogenase; XOD, xanthine oxidoreductase.

As far as we know, ROS modulate the activity of two important glycolytic enzymes, namely glyceraldehyde-3-phosphate dehydrogenase (GAPDH) and PKM2 (**Figure 3**). GAPDH catalyzes the sixth step of glycolysis in which glyceraldehyde-3-phosphate (G-3-P) is converted into 1,3-bisphosphoglycerate. In addition to its glycolytic role, GAPDH has also been described to play a key role in alternative cellular processes such as cytoskeletal dynamics, DNA repair, and cell death (133). GAPDH contains multiple free cysteine thiols, several of which are arranged in the active site of the protein. Among them, Cys-152 has been shown to be exceptionally susceptible to both S-oxidation and S-glutathionylation (134). Pyruvate kinase (PK) catalyzes the last reaction of glycolysis, the conversion of phosphoenolpyruvate and ADP to pyruvate and ATP. There are four isoforms of this enzyme in mammals. While the PKM1 isoform is constitutively active, PKM2 can be regulated. PKM2 can fluctuate between a highly active tetrameric state and a virtually inactive dimer (135). Oxidation of PKM2 Cys-358 promotes formation of the less active dimer (136).

The mitochondrion represents a unique microenvironment for thiol modifications. On the one hand, proton pumping from the matrix to the intermembrane space generates an alkalinized matrix environment, which favors the ionized state of protein cysteine residues in the matrix. On the other hand, given the high ROS production and the elevated concentration of reduced glutathione (GSH) found in the mitochondria, it is very likely that S-glutathionylation of susceptible residues occurs.

The pyruvate dehydrogenase complex (PDH) converts pyruvate to acetyl-CoA, which will enter the TCA cycle. In 1996, Tabatabaie et al. (137) demonstrated that ROS generated in the xanthine oxidase/hypoxanthine complex inactivated PDH by direct oxidation in isolated enzyme assays. On the other hand, PDK2, a negative regulator of the PDH complex, is inhibited by reversible oxidation of Cys-45 and Cys-392 and results in increased PDH activity (138). Inhibition of both PDH and PDK2 by ROS seems somehow contradictory, but at the same time illustrates perfectly why we must discern ROS modifications in their biological context: the same input through two different targets might eventually lead to different outcomes. Metabolic regulation involves several checkpoints. Thus, enhanced TCA cycle feeding induced by PDK2 oxidation could later be counterbalanced by redox regulation of enzymes involved in the TCA cycle, as we discuss below (**Figure 3**).

Among the TCA enzymes, oxidation directly inhibits α-ketoglutarate dehydrogenase (KGDH) (139), isocitrate dehydrogenase 2 (IDH2), and aconitase (140), whereas it activates succinate dehydrogenase (SDH) (141) (**Figure 3**). Other TCA enzymes such as succinyl-CoA synthetase and malate dehydrogenase (140) have cysteine residues that are sensitive to oxidation, but whether this oxidation occurs physiologically and whether it modifies their functions remains to be determined.

Studies have also indicated that ROS are able to inhibit ETC activity by targeting several proteins belonging to complexes I, III, IV, and V (141, 142), thus reducing the flow of electrons through the ETC. In addition, a large variety of enzymes involved in mitochondrial fatty acid oxidation with oxidation-sensitive thiols, and therefore potentially targets of ROS, have been detected (143).

The above-mentioned scenario offers a direct connection between the redox state and the control of the metabolic rate and flow, in which oxidative modifications of metabolic enzymes would appear to be aimed at reducing ROS production. Oxidative inhibition of GAPDH and PKM2 would lead to a rapid accumulation of upstream glycolytic intermediates and a decrease in the downstream glycolytic metabolites. In this way, the flow of carbohydrates would bypass glycolysis and move toward the PPP and amino acid synthesis (136, 141, 142). Activation of these pathways is required to maintain a high rate of cell proliferation, but also to increase NADPH production to alleviate oxidative stress by acting as a cofactor for peroxiredoxin- and glutathione peroxidase-dependent antioxidant pathways. It should also be noted that the effect of ROS on glycolysis may also be influenced by oxidation of HIF1α, AMPK, or ATM pathways that drive glucose metabolism. Moreover, oxidation of proteins involved in the TCA cycle and mitochondrial respiration seems to be aimed at reducing mitochondrial function, which would ultimately reduce the flow of electrons through the ETC and, with it, the production of more ROS that could exceed levels compatible with cell survival. However, as noted above, other modifications such as those of SDH or PDK2 would seem to oppose this idea but could be aimed at supplying substrates to other metabolic pathways. The cellular localization as well as the sensitivity of these enzymes to ROS in each biological context is the key to this contradiction.

The best-known source of ROS in metabolism occurs from “electron leakage” *via* complexes I and III of the ETC. However, there are many other metabolic enzymes that generate ROS at even higher levels (132, 144). Some examples are the mitochondrial flavin adenine dinucleotide-containing dehydrogenases α-glycerophosphate dehydrogenase (mGPDH) and dihydrolipoamide dehydrogenase (a subunit of the KGDH and PDH complexes) (145), which have been mentioned as ROS targets. Additionally, NADPH oxidase–driven ROS production can be activated by certain metabolic enzymes such as PFK2 (118) or 6-phosphogluconate dehydrogenase (146), and by mitochondrial ROS (147), providing another means of enhancing ROS production. In summary, the evidence strongly supports the existence of feedback regulation mechanisms between ROS levels and the metabolic rate (**Figure 3**).

Bearing in mind the bidirectional ROS metabolic regulatory mechanisms, it is appropriate to provide context for this complex landscape in the case of leukemia. Leukemic bulk cells, although heterogeneous and highly flexible, have long been characterized by a prevalent glycolytic metabolism and lower use of mitochondrial metabolism. Likewise, some of the key proteins that regulate flux into the TCA cycle are altered in leukemia. Low aconitase activity (148), overexpression of PDK2 (149) and PKM2 the less active isoform of PKM (150), or relatively frequent IDH mutations (151) result in a slowdown of the TCA cycle. A decrease in activity also occurs with mitochondrial complexes (103). *Per contra*, the main signaling inductors of glycolysis are usually over-activated in leukemia: AMPK (152), ATM (153), and HIF1α (87). When considering that the mentioned enzymes are targets of regulation by oxidation, it is reasonable to formulate the following question: could high ROS levels be the trigger for slowing down the TCA cycle and OXPHOS and promoting glycolysis in this population? In support of this hypothesis, complex II, the unique complex whose oxidation produces an increase in activity (154), is the only one that presents higher activity in AML cells (103). Moreover, LSC that, unlike bulk cells, have low ROS levels, are more dependent on OXPHOS and rely on TCA intermediates and fatty acid oxidation for energy sources (93). In summary, this evidence supports the tight relationship between ROS levels and the metabolic rate in leukemia. The differences between tumor cells and their healthy counterparts make these two aspects appealing therapeutic targets.

## ROS and Metabolism: Leukemic Therapeutic Targets?

For years, the focus of study for cancer treatment had centered on the salient differences between cancerous and healthy conditions—proliferation and metabolism—until the discovery of oncogenes in the 1980s, a finding that shifted the paradigm toward targeted therapy. After 40 years of sustained efforts in developing efficient targeted therapies, there have been limited advances, especially in non-solid tumors. Therapeutic tools such as imatinib, sorafenib, or ibrutinib have reached the market but are only useful for a small percentage of cases. The promising results that immunotherapy is demonstrating in hematological disorders is bringing back broad-spectrum therapies, those that act on the commonalities of malignancies. Going further, most types of leukemia are characterized by very diverse molecular landscapes, and by a high heterogeneity within the leukemic cell population itself (LSC, blast, or bulk) that argues against the simplistic approach of designing generalized monotherapies based on the regulation of a single alteration or mutation. In such a scenario, ROS and metabolism are emerging as interesting therapeutic targets, including targeting of chemoresistant cells (47, 91).

There are a vast number of pharmacological strategies that allow enhancing or decreasing cellular ROS levels. Boosting ROS levels would push tumor cells to a situation of oxidative stress incompatible with cell viability. Upon the same insult, normal cells would not reach such a level of oxidative stress, and, ideally, they would be spared. In addition, researchers have realized the mechanism of action of drugs used long ago in the treatment of hematological malignancies depends on an increase in ROS. Such is the case of arsenic trioxide (As_2_O_3_) (155), used to treat acute promyelocytic leukemia (APL); cytarabine (156), used to treat AML; or bortezomib (157), used to treat multiple myeloma. This scenario has paved the way for the active search and development of novel pro-oxidant anti-tumor strategies. Elevated ROS levels could be achieved by different mechanisms, such as hampering the cellular antioxidant defense or inducing ROS production by NADPH oxidases and mitochondria. Some of these pro-oxidant strategies have reached clinical trials, as we have reviewed recently (12).

On the other hand, if tumor cells depend on high ROS levels for their growth, reducing ROS levels could also be a suitable therapeutic strategy. Therapy based on blocking ROS indiscriminately using antioxidants has not fulfilled the initial expectations of limiting tumor development; in some cases, the opposite has even been suggested. Perhaps the answer to this antioxidant approach actually lies in focusing our efforts on targeting specific ROS-producing sources rather than general modulation. The interest in the NADPH oxidase family as a therapeutic target has increased recently, together with an intense search for specific inhibitors of these enzymes, some of which have already gone into clinical trials (12). Animal models support the requirement of NOX2 for leukemia development (64, 74), and some reports support the feasibility of using NOX2 as target against myeloproliferative neoplasm (73), CML (63, 67), and AML (158). This evidence encourages additional studies in this direction. Although NADPH oxidases seem to be one of the better option for inhibiting ROS production in a specific way, it is important not to overlook other sources of ROS such xanthine oxidoreductase (XOD) (159) or IDH1 (160). More information on the various pro-oxidant and antioxidant alternatives for the treatment of leukemia can be found at (12, 47).

Likewise, targeting metabolism is also emerging due to its efficacy in different types of leukemia as well as its effects on stemness and relapse situations. Targeting metabolism is an interesting approach, considering HSC can remain quiescent if they sense the unavailability of nutrients or cannot use their metabolic routes, while LSC and bulk cells have constitutively active metabolism due to oncogenic signals. As recently reviewed (161, 162), there has been intense efforts in this direction. Targeting enzymes from glycolysis, the TCA cycle, OXPHOS, or lipid or protein metabolism have proven to be promising anti-leukemic approaches, in preclinical and clinical trials. CPI-613, an inhibitor of PDH and KGDH, is one of the most prospective cases because it has just been tested in a phase 3 clinical trial in combination with a high dose of cytarabine and mitoxantrone for relapsed/refractory AML older patients (NCT03504410).

Furthermore, there are already three strategies approved by the Food and Drug Administration in leukemia whose mechanism of actions implies metabolism modulation. The first is asparaginase for ALL treatment in 2006. Asparaginase is medically prescribed for childhood ALL, but is also administered to older patients with ALL, even though it is less efficacious (163). Enasidenib, an IDH2 inhibitor, and ivosidenib, an IDH1 inhibitor, were approved in 2017 and 2019, respectively, for the treatment of patients with AML bearing *IDH2* or *IDH1* mutations. Enasidenib was approved for refractory and relapsing patients after transplant, but it was withdrawn in 2019 because it did not demonstrate sufficient efficacy. Ivosidenib has been approved for patients with *de novo* or refractory disease. Lastly, venetoclax (VENCLEXTA^®^) is approved for the treatment of three hematological malignancies: adult patients with CLL who have received at least one prior therapy; patients with small lymphocytic lymphoma, with or without 17p deletion, who have received at least one prior therapy; and in combination with hypomethylating agents, azacytidine or decitabine, or low-dose cytarabine for the treatment of newly-diagnosed adults with AML who are ≥ 75 years, or who have comorbidities that preclude use of intensive induction chemotherapy. Of note, recent reports support that venetoclax can sensitize AML cells to chemotherapy (164), which may be due to suppression of OXPHOS (165), facilitating LSC elimination. In addition, other leukemia treatment options have been proposed with metabolism as a target, as discussed previously (161, 162).

Immune cells, which collaborate in the eradication of the malignant cells, mostly share metabolic features with cancer cells. Therefore, metabolic therapies should be studied in the context of the microenvironment to avoid immune cell damage (166). Particularly in the case of acute leukemia, the ratio of microenvironment cells to bad cells is low, as it is a dispersed and highly proliferative cancer. Therefore, leukemia is assumed to be one of the most appropriate targets for metabolic targeting.

Another potential application for these treatments is HSC or stem cell transplant. Maintaining reduced ROS levels on par with impeding OXPHOS activation remains essential for guaranteeing HSC quality, survival, and reconstitution ability. Thus, some antioxidant conditions have been tested for HSC extraction and expansion (16, 167), and metabolic manipulation has recently been proposed to achieve the same goal (168, 169).

Considering the accumulated evidence, it is reasonable to think that targeting both ROS production and metabolism could be an effective anti-tumor strategy. In this line, it has been shown that NOX4 inhibition has a strong synergistic effect with the HK inhibitor 2-deoxy-D-glucose in glioblastoma cells (114).

## Conclusion

ROS and metabolism are two cancer hallmarks which are increasingly being considered in leukemia study. Both features have been shown to influence the development, progression, and maintenance of these diseases. Thus, redox signaling and metabolism have been targeted with promising results. In this review, we have described the bidirectional connection that exists between ROS and metabolism, which may be governing leukemogenesis and leukemic cell function. In light of the scientific evidence presented, we propose to treat leukemia by combining ROS and metabolism strategies. The feasibility of this approach should be tested in the near future.

## Author Contributions

MR-G, CI, and ÁH-H, contributed to conception and writing of the manuscript. MR-G and CI equally contributed to this work, therefore, they should be considered co-first authors. All authors contributed to manuscript revision, read, and approved the submitted version.

## Conflict of Interest

The authors declare that the research was conducted in the absence of any commercial or financial relationships that could be construed as a potential conflict of interest.

## Publisher’s Note

All claims expressed in this article are solely those of the authors and do not necessarily represent those of their affiliated organizations, or those of the publisher, the editors and the reviewers. Any product that may be evaluated in this article, or claim that may be made by its manufacturer, is not guaranteed or endorsed by the publisher.

## Abbreviations

ALL, Acute lymphocytic leukemia; AML, Acute myeloid leukemia; AMPK, AMP-activated protein kinase; APL, Acute promyelocytic leukemia; BM, Bone marrow; CML, Chronic myeloid leukemia; CLL, Chronic lymphocytic leukemia; DALYs, disability-adjusted life years; ERK, Extracellular signal-regulated kinase; ETC, Electron transport chain; G-3-P, Glyceraldehyde-3-phosphate; G6PD, Glucose-6-phosphate dehydrogenase; GAPDH, Glyceraldehyde-3-phosphate dehydrogenase; H_2_O_2_, Hydrogen peroxide; HK, Hexokinase; HSC, Hematopoietic stem cells; IDH, Isocitrate dehydrogenase; KGDH, α-ketoglutarate dehydrogenase; LKB1, Liver kinase B1; LSC, Leukemic stem cells; MAPK, Mitogen-activated protein kinase; MEK, Mitogen-activated protein kinase kinase; mGPDH, Mitochondrial α-glycerophosphate dehydrogenase; MPP, Multi-potent progenitor cells; mtDNA, Mitochondrial DNA; mTOR, Mammalian target of rapamycin; OXPHOS, Oxidative phosphorylation; PDH, Pyruvate dehydrogenase complex; PDK, Pyruvate dehydrogenase kinase; PFK2, Phosphofructokinase 2; PFKFB3, 6-phosphofructo-2-kinase/fructose-2,6-bisphosphatase; PI3K, Phosphoinositide 3-kinase; PK, Pyruvate kinase; PPP, Pentose phosphate pathway; PTPMT1, Protein tyrosine phosphatase mitochondrial 1; ROS, Reactive oxygen species; SDH, Succinate dehydrogenase; TCA, Tricarboxylic acid; T-ALL, T-cell acute lymphocytic leukemia.
